# Dermatological Diseases and Household Behavioral Spillovers: A Conceptual Framework

**DOI:** 10.3390/healthcare14193173

**Published:** 2026-09-24

**Authors:** Yuval Arbel, Netanel Kerner, Oriya Kerner, Miryam Kerner

**Affiliations:** 1 Sir Harry Solomon School of Economics and Management, Western Galilee College, Akko 2412101, Israel; 2 Faculty of Medicine, Hebrew University of Jerusalem, Jerusalem 9112102, Israel; netanel.kerner@mail.huji.ac.il; 3 The Ruth and Bruce Rapoport Faculty of Medicine, Technion–Israel Institute of Technology, Haifa 3109601, Israel; oriya.kerner@campus.technion.ac.il (O.K.); drkernerm@gmail.com (M.K.); 4 Kaplan Medical Center, Rehovot 7661041, Israel

**Keywords:** dermatology, behavioral spillovers, household behavior, behavioral adaptation, health economics, coordination game

## Abstract

**Background:** Chronic diseases may generate behavioral responses extending beyond the affected individual. Although family health effects and behavioral spillovers have been examined in health economics and behavioral research, their relevance to dermatological disease management has received limited conceptual attention. **Objective:** To develop a conceptual framework distinguishing individual behavioral adaptation from household behavioral spillovers and identifying the conditions under which disease management behaviors may extend beyond the patient. **Methods:** This conceptual study integrates theoretical and empirical literature from health economics, behavioral science, family health research, and dermatology. Four dermatological conditions—photosensitivity disorders, atopic dermatitis, psoriasis, and acne—are used as purposively selected illustrations of contrasting behavioral mechanisms. A coordination-game model is additionally developed to formalize one mechanism of interdependent patient–household behavioral adaptation. **Results:** The framework proposes two analytically distinct but interrelated processes. Individual behavioral adaptation is hypothesized to depend on trigger clarity, behavioral controllability, and feedback immediacy, whereas the translation of disease management behavior into household spillovers additionally depends on behavioral visibility, environmental sharedness, and household relevance. Patient and household adaptation may be reciprocal, particularly in caregiver-mediated disease management. The coordination game demonstrates how strategic complementarity can generate both coordinated-adoption and non-adoption equilibria when the benefits of behavioral adaptation depend on compatible household behavior. Behavioral spillovers are treated as directionally neutral and may generate positive, negative, mixed, or null health and welfare consequences. **Conclusions:** The proposed framework provides testable propositions for future longitudinal, dyadic, and household-level research on disease-related behavioral adaptation. More broadly, although dermatological diseases provide the illustrative setting, the framework may be applicable to other chronic diseases in which management affects household behavior; incorporating effects on patients and their close social environment may improve resource allocation and provide a more complete basis for evaluating aggregate welfare.

## 1. Introduction

Chronic diseases influence health systems through mechanisms that extend beyond their direct clinical consequences. In addition to generating healthcare expenditures and productivity losses, chronic conditions may induce behavioral responses affecting not only patients themselves but also their families and immediate social environment. Health economics has long recognized that health-related decisions may generate consequences beyond the individual decision maker [1], while more recent research has examined family health behaviors and behavioral responses to health events. Importantly, however, interpersonal behavioral spillovers should not automatically be equated with economic externalities, particularly within households, where caregiving, shared preferences, joint resource constraints, and collective decision-making may internalize some of the resulting benefits and costs. Despite growing interest in such interpersonal effects, relatively little attention has been devoted to developing a framework explaining when disease management behaviors extend beyond the affected individual and influence household behavior.

Behavioral spillovers, as conceptualized in this article, refer to changes in the behavior of other household members arising in connection with a patient’s disease or disease management behavior. Such changes may occur through shared environments, observational learning, social interaction, caregiving, coordinated action, or common daily routines. Importantly, behavioral spillover is conceptually distinct both from the patient’s own behavioral adaptation and from the health or welfare consequences ultimately caused by behavioral diffusion. Household behavioral responses may generate positive, negative, mixed, or null consequences, and whether they constitute economic externalities additionally depends on whether the resulting benefits or costs are internalized within the relevant decision-making process.

Dermatological diseases provide a particularly informative setting for examining these processes. Skin disorders frequently require repeated disease management behaviors integrated into everyday routines and may involve interactions with environmental or lifestyle factors. Preventive and management practices such as photoprotection, skin-care routines, treatment adherence, modification of relevant exposures, or changes in products used within the home may be visible to other household members or occur within shared environments. Dermatological conditions also provide substantial heterogeneity in the identifiability of relevant triggers, the extent to which behavioral modification contributes to disease management, the timing and perceptibility of feedback, and the involvement of caregivers or other household members. This variation provides a useful setting for examining why some disease management behaviors may remain predominantly individual whereas others may become embedded in household practices.

Importantly, these characteristics should not be interpreted as fixed properties of particular diagnoses. Behavioral responses depend not only on objective clinical characteristics but also on patients’ perceptions, knowledge, health literacy, previous experience, disease severity, clinician communication, socioeconomic resources, household composition, and caregiving arrangements. Similarly, the extent to which disease management behavior becomes relevant to other household members may depend on whether that behavior is visible, occurs within a shared environment, or is perceived as applicable or beneficial by others. A framework for household behavioral spillovers therefore requires a distinction between the mechanisms governing individual behavioral adaptation and those governing interpersonal or household diffusion.

The present article addresses this gap by developing a conceptual framework comprising two analytically distinct but interrelated processes. At the individual level, trigger clarity, behavioral controllability, and feedback immediacy are proposed as interrelated determinants of behavioral adaptation. At the household level, behavioral visibility, environmental sharedness, and household relevance are proposed as conditions influencing whether disease management behavior extends beyond the patient. The framework additionally recognizes that patient and household adaptation may be reciprocal, particularly in caregiver-mediated disease management, so that household behavior may precede, facilitate, reinforce, or inhibit patient-level adaptation. Four dermatological conditions—photosensitivity disorders, atopic dermatitis, psoriasis, and acne—are used as purposively selected illustrative examples providing contrasting applications of these mechanisms rather than as empirically established rankings of behavioral-spillover potential.

A coordination-game model is additionally developed to formalize one mechanism through which patient and household behavioral adaptation may become interdependent. When the effectiveness or net benefit of behavioral adaptation by one party depends on compatible behavior by another household member, strategic complementarity may generate both coordinated-adoption and non-adoption equilibria. The model therefore illustrates why mutually beneficial household adaptation may not arise automatically even when coordinated behavior would improve outcomes for both parties. The coordination game is intended as one possible mechanism within the broader framework rather than as a general description of all forms of household behavioral diffusion.

Accordingly, the central research question addressed in this article is:

**How can differences in disease characteristics, patient behavioral adaptation, and household context generate different patterns of household behavioral spillover?**

By addressing this question, the article contributes to health economics, behavioral, and dermatology literature in three ways. First, it distinguishes individual behavioral adaptation from household behavioral diffusion and identifies conceptually separate determinants of each process. Second, it clarifies the relationship between behavioral spillovers and economic externalities while allowing the health and welfare consequences of household behavioral responses to be positive, negative, mixed, or null. Third, it develops a theoretically structured and empirically testable account of reciprocal patient–household adaptation, including a formal coordination-game mechanism, which can guide future longitudinal, dyadic, and household-level research.

Although dermatological diseases provide the illustrative setting, the proposed mechanisms may also apply to other diseases when disease management affects shared routines, caregiving, household behavior, or the health of individuals beyond the treated patient. This broader perspective is consistent with health–economic research demonstrating that disease-control interventions can generate health effects extending beyond directly treated or protected individuals. For example, Claypool et al. [2] quantify positive health externalities associated with interventions targeting chikungunya and dengue. Although such population-level externalities are conceptually distinct from the household behavioral spillovers examined here, they reinforce the broader principle that evaluating disease management solely at the level of the individual patient may omit relevant effects on others. From a health–economic perspective, incorporating consequences experienced within the patient’s immediate social environment may therefore provide a more complete basis for resource allocation and aggregate welfare assessment, although generalizability beyond dermatology requires empirical testing.

## 2. Framework Development and Selection of Illustrative Conditions

This study adopts a conceptual rather than systematic review methodology. The framework was developed through an iterative synthesis of theoretical and empirical literature from health economics, behavioral science, family health research, and dermatology. Established behavioral theories were used to identify mechanisms relevant to individual behavioral adaptation, while the family health and health economics literature was used to distinguish patient-level adaptation from interpersonal and household-level behavioral diffusion. The resulting constructs were refined to separate determinants of individual adaptation—trigger clarity, behavioral controllability, and feedback immediacy—from conditions facilitating household diffusion—behavioral visibility, environmental sharedness, and household relevance.

The four dermatological examples were selected purposively rather than epidemiologically. They were chosen because they provide contrasting illustrations of the mechanisms proposed in the framework, including differences in the identifiability of relevant exposures, the role of behavioral modification in disease management, the timing and perceptibility of feedback, the extent to which management behaviors occur in shared environments, and the involvement of caregivers or other household members. The selected conditions are therefore not intended to represent the full spectrum of dermatological disease or to constitute an empirically derived ranking.

Photosensitivity disorders are included as a mechanism-based category because sensitivity to ultraviolet exposure provides a particularly transparent illustration of the relationship between identifiable environmental exposure and preventive behavior. Atopic dermatitis provides an example in which caregivers and household environmental practices may play an important role; psoriasis illustrates a multifactorial condition in which behavioral modification represents only one component of disease management; and acne illustrates a condition in which substantial treatment-related behavioral adaptation may remain predominantly individual. These contrasts are used solely to generate and clarify theoretical propositions. They should not be interpreted as evidence that patients with a given diagnosis occupy fixed positions on the proposed dimensions.

The supporting literature was identified iteratively for its relevance to the conceptual mechanisms under consideration rather than through a protocol designed to estimate effect sizes or exhaustively identify all publications. Accordingly, the literature review should be interpreted as a theory-building synthesis rather than a systematic or scoping review.

## 3. Behavioral Adaptation and Household Spillovers: Theoretical and Empirical Foundations

### 3.1. Individual Health Behavior and Behavioral Adaptation

Health-related behavior is shaped by the interaction of perceived health risks, expected benefits and costs of action, perceived control, social influences, and feedback from previous behavioral choices. Established theories of health behavior therefore provide a useful foundation for understanding why a diagnosis or disease management recommendation may lead some patients to modify their behavior more extensively than others.

The Health Belief Model emphasizes perceived susceptibility, perceived severity, perceived benefits and barriers, and self-efficacy as determinants of health-related behavior [3]. Similarly, the Theory of Planned Behavior identifies attitudes, subjective norms, and perceived behavioral control as determinants of behavioral intentions and subsequent behavior [4]. Social Cognitive Theory places particular emphasis on self-efficacy, outcome expectations, environmental facilitators and impediments, and the reciprocal relationships among individuals, behavior, and their environments [5]. Together, these approaches indicate that behavioral adaptation cannot be inferred solely from the objective characteristics of a disease. What matters behaviorally is also how patients perceive the disease, their capacity to identify relevant actions, their expectations regarding the consequences of those actions, and their perceived ability to implement them.

These theoretical traditions provide the foundation for, but are not identical to, the dimensions proposed in the present study. Specifically, the framework developed below organizes individual behavioral adaptation around three interrelated constructs: **trigger clarity**, **behavioral controllability**, and **feedback immediacy**. Trigger clarity concerns the extent to which patients can identify environmental or behavioral factors associated with symptom onset or exacerbation. Behavioral controllability concerns the perceived or clinically expected extent to which recommended behavioral modification can contribute to disease management. Feedback immediacy concerns how rapidly and perceptibly the consequences of behavioral modification become apparent. These three constructs are proposed here as a new conceptual synthesis rather than as a reproduction of an established behavioral model.

The relationship between these constructs should not be interpreted as necessarily additive or independent. For example, the behavioral significance of a clearly identifiable trigger may depend on whether the patient perceives an effective means of avoiding or modifying exposure. Likewise, rapid feedback may reinforce a behavioral response more strongly when the patient can attribute the observed outcome to a particular action. This interactive interpretation is consistent with broader behavioral theories in which self-efficacy, outcome expectations, perceived control, and environmental conditions jointly shape behavioral adaptation [4,5].

### 3.2. From Individual Behavior to Household Behavioral Diffusion

Individual behavioral adaptation does not necessarily remain confined to the individual. Health-related behaviors take place within social environments in which other people may observe, facilitate, discourage, imitate, or participate in them. Social Cognitive Theory explicitly recognizes the role of environmental influences and observational processes in behavioral change and emphasizes reciprocal relationships between individuals and their social environments [5].

The household represents a particularly important environment for such processes because family members frequently share physical environments, resources, routines, purchasing decisions, meals, leisure activities, and caregiving responsibilities. The Family Health Climate framework conceptualizes the family environment as a set of shared perceptions and cognitions concerning health behavior that develops through everyday family interactions and experiences. Niermann et al. [6], for example, developed the Family Health Climate Scale to capture the family-level environment surrounding health-related behaviors, emphasizing that health behavior is embedded within the broader family system rather than determined exclusively at the individual level.

This literature suggests an important distinction between the determinants of **individual behavioral adaptation** and the conditions governing **interpersonal behavioral diffusion**. A patient may clearly recognize a trigger, perceive substantial behavioral control, and receive rapid feedback from behavioral modification while nevertheless adopting a behavior that remains entirely personal. Conversely, a behavioral response involving products, routines, or environmental conditions shared by the household may directly involve other household members.

The framework developed in this article therefore proposes three additional constructs relevant to household diffusion: **behavioral visibility**, **environmental sharedness**, and **household relevance**. Behavioral visibility refers to the extent to which disease management behavior is observable to other household members and can therefore become an object of discussion, imitation, reinforcement, or resistance. Environmental sharedness refers to the extent to which the behavior concerns a physical or social environment shared with other household members. Household relevance refers to whether the behavior is applicable, feasible, or perceived as useful by those other members. As with the individual-level constructs, these dimensions represent a conceptual synthesis proposed in this article rather than an established three-variable model.

### 3.3. Family Health Spillovers, Co-Adaptation, and Economic Externalities

Health economics provides additional reasons for treating the family as a relevant unit of behavioral analysis. Traditional models of health investment focus primarily on individual choices, but subsequent work has explicitly recognized that health may also be produced within families. Jacobson [7], extending the Grossman health-capital framework, models family members as contributing to the production of both their own health and the health of other family members. Health-related information, resource constraints, prices, and transfers can therefore influence not only individual health investment but also its distribution within the family.

Empirical research provides evidence that health events affecting one family member can alter the behavior of others. Fadlon and Nielsen [8], using administrative data and health shocks occurring within families, found that spouses and adult children changed preventive-care use and other health-related behaviors following a family member’s health shock. Their findings point particularly to the salience of a relative’s health event as a mechanism influencing subsequent behavior. Hodor [9] similarly distinguishes behavioral and learning channels through which health shocks experienced by one family member may affect healthcare consumption by another. Using evidence from the RAND Health Insurance Experiment, the study identifies both behavioral spillovers and learning effects in preventive health investment.

These findings provide considerably more direct support for household health spillovers than associations between disease status and health behavior measured only at the individual level. They also indicate that behavioral responses need not follow a simple sequence in which a patient first changes behavior and family members subsequently imitate that change. Household members may respond directly to the salience of a diagnosis, acquire information through another family member’s experience, participate in disease management, or alter shared routines. In caregiver-dependent populations, household adaptation may even precede meaningful patient-level behavioral adaptation.

At the same time, **behavioral spillovers should not be treated as synonymous with economic externalities**. Behavioral diffusion describes a change in one person’s behavior associated with the health condition or disease management behavior of another. Whether the resulting effects constitute economic externalities is a separate question. Within households, decisions may incorporate altruism, caregiving, shared preferences, joint production of health, common resource constraints, and collective decision-making. Jacobson’s [7] family health-production model illustrates precisely why health investments affecting several family members cannot automatically be interpreted as effects external to household decision-making.

The distinction is also relevant beyond household and dermatological settings. Claypool et al. [2], for example, model positive health externalities arising from disease-control interventions for chikungunya and dengue, illustrating how the benefits of health interventions may extend to individuals other than those directly targeted. Their analysis concerns population-level health externalities rather than household behavioral diffusion, but it provides a useful complementary example of the broader health–economic principle underlying the present framework: disease management may generate consequences beyond the focal patient, and those consequences may be relevant to economic evaluation.

Accordingly, the present study uses *behavioral spillover* to refer to the diffusion, transmission, or co-adaptation of health-related behavior across household members. Such spillovers may generate economic externalities, but the presence and direction of an externality require additional assessment of whether benefits or costs imposed on others are internalized within the relevant decision-making process. This distinction is especially important when disease management occurs within families rather than among unrelated individuals.

### 3.4. Direction and Consequences of Behavioral Spillovers

A further distinction concerns the direction of the consequences produced by behavioral diffusion. Behavioral spillovers should not be assumed to be beneficial simply because the behavior originated in disease management. Family responses to a health event may alter preventive behavior and health investment, as demonstrated by Fadlon and Nielsen [8] and Hodor [9], but the existence of behavioral transmission does not by itself establish that all consequences are welfare-enhancing.

Household adaptation may generate **positive consequences** when it promotes useful preventive behavior, facilitates adherence, reduces relevant exposures, or improves the household environment. It may generate **negative consequences** when behavioral adaptation becomes unnecessarily restrictive, costly, anxiety-driven, or burdensome. **Mixed consequences** may arise when a behavioral change benefits the patient while imposing financial, psychological, or lifestyle costs on other household members. Finally, behavioral diffusion may produce **null health consequences** if behavior changes without measurable effects on health or welfare.

The conceptual distinction is therefore between the *occurrence of behavioral diffusion* and the *consequences of that diffusion*. The former is the central behavioral outcome considered in this study; the latter remains an empirical question. This directionally neutral interpretation avoids defining successful behavioral spillover by its consequences and makes it possible for subsequent empirical research to test separately whether diffusion occurs and whether its effects are beneficial, adverse, mixed, or negligible.

### 3.5. Why Dermatological Diseases?

Dermatological diseases provide a useful setting in which to examine these mechanisms because disease management frequently involves repeated behavioral decisions integrated into everyday routines. Depending on the condition and patient, management may involve photoprotection, skin-care routines, topical treatment, avoidance or modification of relevant exposures, lifestyle modification, or changes in products used within the home. These behaviors differ substantially in their visibility, their connection to shared environments, and their relevance to other household members. Comparable reliance on environmental modification is also found in other chronic conditions, such as asthma, where environmental control is an established component of management [10].

Importantly, dermatological conditions also differ in the extent to which behavior contributes to disease management. Some conditions have relatively identifiable environmental triggers and preventive responses, whereas others have complex genetic, immunological, inflammatory, and environmental determinants for which behavioral modification represents only one component of management. The proposed framework therefore does not assume that dermatological diseases are inherently more behaviorally controllable than other chronic conditions. Rather, their heterogeneity provides useful conceptual variation for examining the mechanisms through which individual adaptation may or may not extend to the household.

The dermatological setting is also useful because several forms of disease management require sustained adherence. In acne, for example, treatment adherence is itself a substantial management challenge. A systematic review by Snyder et al. [11] found considerable variation in adherence and identified factors including side effects, age, and forgetfulness as important barriers. These findings support treating behavioral adaptation as variable rather than assuming that the existence of a recommended behavior necessarily leads to its implementation.

Likewise, lifestyle modification has been investigated in psoriasis, but the evidence illustrates why behavioral controllability should be defined cautiously. A Cochrane review identified trials of dietary intervention, exercise, and health education, with some evidence of improvement in psoriasis severity and related outcomes, particularly among people with obesity, while also emphasizing limitations in the quality and scope of the available evidence [12]. Thus, behavioral modification may contribute to disease management without implying that psoriasis itself is primarily or uniformly under behavioral control.

### 3.6. Illustrative Dermatological Conditions

The present study uses four dermatological conditions as illustrative applications of the framework: photosensitivity disorders, atopic dermatitis, psoriasis, and acne. They are not intended to constitute a representative epidemiological sample of dermatological disease, nor are they used as empirical evidence validating the proposed framework. Rather, they were selected because their management provides contrasting examples of the potential relationships among environmental triggers, behavioral modification, feedback, individual routines, and household involvement.

#### 3.6.1. Photosensitivity Disorders

Photosensitivity disorders provide an illustrative setting in which environmental exposure may be comparatively identifiable. Ultraviolet radiation can constitute an important trigger in photosensitive conditions, and management may therefore include sunscreen use, protective clothing, and modification of sun exposure. Existing evidence documents photoprotective practices among patients with photosensitive conditions such as systemic lupus erythematosus, while randomized and observational evidence supports the preventive value of sustained photoprotection for relevant skin outcomes [13,14,15].

From the perspective of the present framework, photosensitivity is useful because photoprotective behaviors can be observable and may occur during shared activities. However, the category is heterogeneous, and neither trigger clarity nor feedback immediacy should be treated as uniformly high across all photosensitivity disorders, outcomes, or patients. The example is therefore intended to illustrate a potential combination of relatively identifiable environmental exposure and preventive behavior rather than to assign a fixed ranking to all photosensitive conditions.

#### 3.6.2. Atopic Dermatitis

Atopic dermatitis provides a different illustration because disease management may involve patients, parents, caregivers, and the household environment. Previous research has documented substantial effects of childhood atopic dermatitis on families and caregivers [16], while clinical management may involve skin-care practices and modification of relevant exposures [17]. In young children in particular, many management behaviors are implemented by parents rather than independently by the patient.

Atopic dermatitis therefore illustrates why household adaptation cannot always be conceptualized as a downstream consequence of prior patient adaptation. Caregivers may initiate treatment routines, select products, or modify the child’s environment before the patient is capable of independent disease management behavior. At the same time, triggers and treatment responses in atopic dermatitis are heterogeneous, so household modification should not be assumed to be universally necessary or beneficial. The condition is used here primarily to illustrate the importance of caregiver-mediated behavior, environmental sharedness, and reciprocal patient–household adaptation.

#### 3.6.3. Psoriasis

Psoriasis provides a useful contrast because its pathophysiology is complex and behavioral modification represents only one component of disease management. Lifestyle factors including obesity, smoking, alcohol consumption, physical inactivity, and diet have been examined in relation to psoriasis, and interventions involving dietary change and exercise have shown some potential benefits. However, a Cochrane review concluded that the available evidence for lifestyle interventions varies in quality and remains incomplete across several relevant behaviors [12]. Psoriasis also imposes substantial quality-of-life and economic burdens [18,19], although these aspects of disease burden are analytically distinct from the extent to which behavioral modification can influence disease management.

Within the present framework, psoriasis therefore illustrates a setting in which potentially relevant behavioral adaptations exist but their connection with immediate disease feedback may be less straightforward than in situations involving a clearly identifiable exposure and preventive response. This is a conceptual comparison rather than an assertion that patients with psoriasis necessarily have low behavioral controllability or limited household spillover.

#### 3.6.4. Acne

Acne illustrates a condition in which behavioral adaptation may be substantial while many management behaviors remain primarily individual. Treatment may involve repeated topical or oral medication, skin-care practices, and other patient-level routines. Evidence from systematic reviews demonstrates that adherence to acne treatment varies and is influenced by factors such as side effects, forgetfulness, treatment complexity, inconvenience, knowledge, and patient–clinician communication [11]. Acne is also highly prevalent worldwide [20]; however, prevalence alone does not imply that disease management behaviors will diffuse within the household.

The importance of acne for the present framework is therefore not that it is assumed to generate no household spillover. Rather, it provides an example in which substantial individual adaptation may coexist with comparatively fewer opportunities for diffusion when disease management behaviors are personal, privately performed, or of limited relevance to other household members. This illustrates the central distinction between determinants of individual behavioral adaptation and determinants of household behavioral diffusion (Table 1).

### 3.7. Literature Gap and Conceptual Contribution

The preceding literature provides important but fragmented insights into health behavior, family health production, behavioral transmission within families, and behavioral management of dermatological disease. Established behavioral theories explain how perceptions of control, self-efficacy, expected outcomes, social influences, and environmental conditions shape individual behavior [3,4,5]. Family level research demonstrates that health behavior is embedded within shared family environments [6], while health economics studies provide evidence that health events experienced by one family member can influence the health investments and behavior of others [7,8,9].

What remains less developed is an integrated conceptual framework distinguishing the factors that shape **a patient’s own behavioral adaptation** from those determining whether disease management behavior becomes relevant to and diffuses among **other household members**. This distinction is important because strong individual adaptation need not generate household spillover, while caregiver or household adaptation may sometimes precede meaningful patient-level adaptation.

The present study addresses this gap by proposing a two-stage conceptual architecture. At the individual level, **trigger clarity, behavioral controllability, and feedback immediacy** are proposed as interrelated determinants of behavioral adaptation. At the household level, **behavioral visibility, environmental sharedness, and household relevance** are proposed as conditions affecting whether behavioral responses extend beyond the patient. The framework additionally permits reciprocal patient–household relationships and distinguishes behavioral diffusion from its subsequent consequences, which may be positive, negative, mixed, or null. These constructs are intended to organize and generate empirically testable hypotheses rather than to represent disease-specific effects already established in the literature.

## 4. A Conceptual Framework for Individual Behavioral Adaptation and Household Behavioral Spillovers

### 4.1. Conceptual Foundations

The literature reviewed in the previous section suggests that dermatological diseases may differ not only in their clinical characteristics but also in the behavioral responses associated with their management. The framework developed here seeks to organize these differences conceptually rather than to establish empirically that particular diseases generate stronger or weaker behavioral spillovers. Its purpose is to distinguish two related but analytically separate processes: **individual behavioral adaptation and household behavioral diffusion.**

Individual behavioral adaptation refers to changes in the patient’s own health-related behavior in response to disease diagnosis, symptoms, treatment recommendations, or perceived environmental triggers. Household behavioral spillover refers to changes in the behavior of other household members that arise in connection with the patient’s disease management. Although the two processes may be related, individual adaptation does not necessarily produce household spillover. A behavioral change may remain entirely individual when it is not visible, relevant, or applicable to other household members. Conversely, particularly where children or dependent patients are concerned, household members or caregivers may initiate behavioral changes before meaningful patient-level adaptation occurs.

The framework therefore distinguishes between determinants of **individual behavioral adaptation** and **determinants of interpersonal or household diffusion**. Three dimensions—trigger clarity, behavioral controllability, and feedback immediacy—are proposed as determinants of the first process. A second set of characteristics—behavioral visibility, environmental sharedness, and household relevance—is proposed to influence whether behavioral responses extend beyond the patient.

Importantly, these constructs should not be interpreted as fixed properties of particular diagnoses. Their behavioral relevance may depend on both objective clinical characteristics and patients’ perceptions of those characteristics. Knowledge, health literacy, clinician communication, previous experience, socioeconomic resources, disease severity, and other individual circumstances may influence how clearly a patient perceives a trigger, how controllable disease management appears, and how clinical feedback is interpreted. Accordingly, the classification of the dermatological conditions considered in this article represents expected conceptual tendencies rather than fixed disease-specific rankings.

### 4.2. Stage I: Individual Behavioral Adaptation

The first stage of the framework concerns the mechanisms that may encourage patients to initiate and maintain disease-related behavioral changes. Three interrelated dimensions are proposed: **trigger clarity (TC), behavioral controllability (BC), and feedback immediacy (FI).**

**Trigger clarity** refers to the extent to which relevant environmental or behavioral factors associated with symptom onset or exacerbation can be identified by the patient. Trigger clarity may reflect clinically established disease characteristics, patient knowledge, or the patient’s interpretation of the relationship between exposures and symptoms. Where triggers are relatively identifiable, patients may find it easier to connect specific preventive behaviors with disease management. Where triggers are multiple, uncertain, or difficult to distinguish, behavioral responses may be less consistent.

**Behavioral controllability** refers to the perceived or clinically expected extent to which recommended behavioral modification can influence exposure, symptoms, or disease management. The concept does not imply that the disease itself is under the patient’s control or that patients are responsible for disease activity. Rather, it concerns the scope for behavior to contribute to management alongside the biological, genetic, immunological, and pharmacological determinants of the condition.

**Feedback immediacy** refers to the temporal proximity and perceptibility of the relationship between behavioral modification and subsequent clinical or experiential outcomes. When patients perceive relatively rapid changes following a behavioral modification, reinforcement of that behavior may be stronger. When feedback is delayed, inconsistent, or difficult to attribute to the behavioral change, sustained adaptation may be less likely.

Individual behavioral adaptation may therefore be represented conceptually as:

(1) $$BA=f\left(TC,BC,FI\right)$$

where

BA = expected intensity of individual behavioral adaptation;

TC = trigger clarity;

BC = behavioral controllability; and

FI = feedback immediacy.

The function $f\left(\cdot \right)$ represents a general conceptual relationship rather than a specified statistical or econometric model. In particular, Equation (1) does not assume that the three dimensions are independent or that their effects are necessarily additive or multiplicative. They may interact. For example, immediate feedback may provide stronger behavioral reinforcement when the patient can clearly identify the trigger and understand which behavioral modification produced the observed response.

Subject to these qualifications, the framework proposes the following directional relationships:

(2) $$\frac{\partial BA}{\partial TC}>0,\frac{\partial BA}{\partial BC}>0,\frac{\partial BA}{\partial FI}>0.$$

These relationships should be understood as **testable theoretical propositions**, not as empirically established effects. They imply that, other things being equal, greater perceived trigger clarity, greater scope for behavioral modification to contribute to disease management, and more immediate observable feedback are expected to be associated with stronger individual behavioral adaptation.

### 4.3. Stage II: From Individual Adaptation to Household Behavioral Spillovers

Individual behavioral adaptation alone is insufficient to explain household behavioral spillovers. A patient may adopt a behavior consistently without that behavior affecting anyone else. The second stage of the framework therefore considers the conditions under which patient-level behavioral adaptation may extend to other household members.

Three broad characteristics are proposed to facilitate this process: **behavioral visibility (BV), environmental sharedness (ES), and household relevance (HR).**

**Behavioral visibility** refers to the extent to which disease management behaviors are observable to other household members. Behaviors that are repeatedly performed in shared settings may provide greater opportunities for observational learning, imitation, discussion, or reinforcement than behaviors undertaken privately.

**Environmental sharedness** refers to the extent to which disease management behavior concerns a physical or social environment shared with other household members. Changes involving household products, cleaning practices, sun exposure during family activities, food purchasing, or other common routines may directly involve other individuals even when the original motivation for the change arises from one patient’s condition.

**Household relevance** refers to the extent to which a disease management behavior is applicable, feasible, or perceived as useful by other household members. A highly visible behavior need not diffuse if other household members regard it as irrelevant to themselves. Conversely, behaviors perceived as beneficial, convenient, protective, or compatible with existing household routines may be more likely to be adopted more broadly. Household relevance may therefore reflect factors such as caregiving responsibilities, relationship closeness, perceived benefits and costs, household resources, and opportunities for coordinated action.

A patient-mediated pathway of household behavioral spillover can therefore be represented conceptually as:

(3) $$BS=g\left(BA,BV,ES,HR\right),$$

where

BS = expected intensity of household behavioral spillover;

BA = individual behavioral adaptation;

BV = behavioral visibility;

ES = environmental sharedness; and

HR = household relevance.

As with Equation (1), Equation (3) is not intended to impose a particular functional form. It identifies conceptually relevant determinants that can subsequently be operationalized and tested empirically. Most importantly, it does **not** imply that greater individual behavioral adaptation automatically produces greater household spillover. The relationship between BA and BS is conditional on whether the behavior is visible, occurs within a shared environment, and has relevance to other household members.

This distinction helps explain why diseases or disease management practices associated with substantial individual behavioral adaptation need not produce correspondingly large household effects. A patient may adhere strongly to an individualized skin-care or treatment regimen that has little relevance to other family members. Conversely, a behavioral modification involving the shared household environment may affect family practices even when patient-level adaptation is limited.

### 4.4. Reciprocal Patient–Household Adaptation

Although Equation (3) describes a patient-mediated pathway, household adaptation should not be conceptualized exclusively as a consequence of prior patient adaptation. The relationship may be **bidirectional**.

This issue is particularly important in the management of children, adolescents, older adults, or other patients who depend on caregivers. For example, in childhood atopic dermatitis, parents may select skin-care products, modify detergents and cleaning routines, manage environmental exposures, or implement treatment recommendations before the child is capable of meaningful independent behavioral adaptation. In such circumstances, household behavioral change may precede, facilitate, or substitute for patient-level adaptation.

The framework therefore allows two pathways:

(4) BA→BS

and

(5) BS→BA.

More generally,

(6) BA↔BS.

The reciprocal relationship also recognizes that household responses may reinforce or weaken patient behavior over time. Supportive household practices may reduce the cost or inconvenience of adherence and reinforce behavioral adaptation. Conversely, household resistance, incompatible routines, financial constraints, or competing preferences may inhibit the patient’s ability to maintain recommended behaviors.

Accordingly, behavioral spillover is better understood as a dynamic process of **patient–household co-adaptation** than as a strictly sequential transition from patient behavior to household behavior.

### 4.5. A Coordination Game of Patient–Household Behavioral Adaptation

The reciprocal relationship between patient and household behavior described above may, in some circumstances, involve strategic coordination. In particular, some disease management behaviors may be more effective, less burdensome, or easier to sustain when the patient and other household members adopt compatible practices. Environmental modifications, changes in shared household products, and preventive behaviors undertaken during joint activities provide possible examples. In such settings, the net benefit of adoption to one party may depend partly on whether the other party also adopts.

This interaction can be represented as a simple two-player coordination game. The two players are the patient (P) and the household (H), and each chooses between two strategies: **Adopt** (A) or **Not Adopt** (N) the relevant preventive behavior. For simplicity, consider first a symmetric version of the game.

Let B>0 denote the benefit received by each player when the preventive behavior is implemented through coordinated adoption, and let C>0 denote the individual cost of adoption. The cost may represent monetary expenditure, time, inconvenience, restrictions on routine, or other burdens associated with implementing the behavior. If one player adopts while the other does not, the adopting player incurs the cost C, but receives only a fraction αB of the benefit available under coordinated adoption, where 0≤α<1. The parameter α therefore captures the effectiveness of unilateral relative to coordinated adoption.

The resulting interaction is shown in Table 2.

The interaction constitutes a coordination game when

(7) αB<C<B.

The first inequality,

αB<C,

implies that unilateral adoption is not individually worthwhile. If the other player does not adopt, the adopting player obtains

αB−C<0,

and therefore prefers not to adopt. The second inequality,

C<B,

implies that coordinated adoption is individually worthwhile. If the other player adopts, adoption produces

B−C>0,

whereas non-adoption produces zero. Thus, each player’s optimal strategy depends on the behavior of the other player.

Under these conditions, the game has **two pure-strategy Nash equilibria**:

$$\left(A,A\right)\mathrm{and}\left(N,N\right).$$

At $\left(A,A\right)$, neither player has an incentive to deviate unilaterally because B−C>0. If either player switched from adoption to non-adoption while the other continued to adopt, that player’s payoff would fall from B−C to zero. Joint adoption is therefore a Nash equilibrium.

At $\left(N,N\right)$, neither player has an incentive to adopt unilaterally because αB−C<0. A unilateral deviation from non-adoption to adoption would reduce that player’s payoff from zero to a negative value. Joint non-adoption is therefore also a Nash equilibrium.

The existence of these two equilibria illustrates the coordination problem. Although both players may be better off under coordinated adoption than under coordinated non-adoption, the socially and individually preferable outcome need not arise automatically. If each player expects the other not to adopt, non-adoption is individually rational and the $\left(N,N\right)$ equilibrium may persist. Conversely, when each expects the other to adopt, coordinated adoption becomes individually rational. The model therefore exhibits **strategic complementarity**: the incentive for one player to adopt increases when the other player is expected to adopt.

### 4.6. Scope and Limitations

Several limitations of both the broader framework and the coordination model should be acknowledged. First, the framework is conceptual and does not quantify behavioral spillovers or establish causal relationships. The dermatological conditions considered in this article are illustrative cases selected to clarify theoretical mechanisms rather than to provide a representative epidemiological sample or definitive ranking of diseases. The proposed dimensions and relationships therefore require validation using observational, longitudinal, experimental, or household-level data. Future empirical applications of the framework should therefore include, where feasible, an appropriate negative or neutral spillover comparator, allowing observed household behavioral changes to be distinguished from background changes that would have occurred in the absence of disease-related behavioral diffusion.

Second, the coordination game formalizes only one possible form of patient–household interaction. It assumes two players, two discrete strategies, symmetric benefits and costs, and a stylized payoff structure. Actual households may contain multiple decision makers with heterogeneous preferences, resources, caregiving responsibilities, information, and constraints, and behavioral adaptation may arise through observational learning, caregiver initiative, shared environments, or social influence without strategic coordination. Accordingly, the game should be interpreted as a theoretical mechanism illustrating strategic complementarity rather than as a complete representation of household decision-making.

Third, although dermatological diseases provide a useful setting for developing the framework, its applicability to other diseases should not be assumed. The underlying logic may extend to other chronic conditions when management has consequences for shared household behavior, but the relevant triggers, costs, benefits, feedback mechanisms, and scope of interpersonal diffusion may differ substantially across diseases and populations. Future empirical research should therefore test both the external validity of the framework and whether incorporating effects on close household members improves estimates of resource use and aggregate welfare relative to patient-only approaches.

### 4.7. Numerical Illustration of the Coordination Game

To illustrate the mechanism numerically, suppose that the benefit of coordinated adoption is B=5, the individual adoption cost is C=2, and unilateral adoption produces only 20% of the coordinated benefit, so that α=0.20. The condition in Equation (7) is then satisfied because

αB=1<C=2<B=5.

Substitution of these values into the general payoff structure gives the numerical illustration in Table 3.

Thus, when both patient and household adopt, each obtains a net payoff of 5−2=3. When only one player adopts, that player obtains $0.20\left(5\right)-2=-1$, while the non-adopting player obtains zero. When neither adopts, both receive zero. The numerical example therefore produces the same two pure-strategy Nash equilibria, $\left(A,A\right)$ and $\left(N,N\right)$, while making transparent the benefit–cost structure underlying the coordination problem.

The coordination game should be interpreted as a **stylized representation of one possible mechanism of household behavioral adaptation**, rather than as a claim that all behavioral spillovers in dermatological disease take the form of strategic coordination. The broader framework developed in this article also allows behavioral diffusion through observational learning, caregiver-mediated behavior, shared environments, and other forms of patient–household co-adaptation. In particular, household adaptation may sometimes precede patient adaptation rather than arise from simultaneous strategic choice. The coordination game is therefore most applicable when the effectiveness or net benefit of one party’s behavioral adaptation depends meaningfully on compatible behavior by the other party.

### 4.8. Behavioral Spillovers and Their Consequences

A further distinction is required between the **occurrence of behavioral spillover** and its **health or welfare consequences**. Behavioral diffusion is directionally neutral. The adoption of a behavior by household members does not in itself establish that the resulting effects are beneficial.

Household behavioral responses may generate **positive, negative, mixed, or null consequences**. Positive consequences may occur when household members adopt preventive practices that improve their own health or facilitate disease management for the patient. Negative consequences may arise when adaptation becomes excessive, costly, unnecessarily restrictive, anxiety-driven, or otherwise burdensome. Mixed consequences are possible when the same behavioral response benefits the patient while imposing financial, psychological, or lifestyle costs on other household members. Finally, behavioral diffusion may occur without producing measurable health consequences.

The conceptual pathway should therefore not terminate automatically in “public health benefits.” Instead, the downstream relationship is represented as:

(8) $$BS\to \left\{\begin{array}{c} \mathrm{Positive}\;\mathrm{consequences}\\ \mathrm{Negative}\;\mathrm{consequences}\\ \mathrm{Mixed}\;\mathrm{consequences}\\ \mathrm{Null}\;\mathrm{consequences} \end{array}\right.$$

This distinction also clarifies the relationship between **behavioral spillovers** and **economic externalities**. Behavioral spillover, as used in this framework, refers to the diffusion, transmission, or co-adaptation of health-related behavior across individuals. It does not necessarily constitute an economic externality. Within households, behavioral decisions may reflect altruism, caregiving responsibilities, shared preferences, collective purchasing, common routines, or joint decision-making. To characterize a resulting effect as an externality therefore requires an additional assessment of whether benefits or costs imposed on another individual are external to, rather than internalized within, the relevant decision-making process.

Thus, behavioral spillover and health externality are analytically related but distinct concepts:

(9) Behavioral Spillover≠Health Externality.

A behavioral spillover may generate an externality, but the existence and direction of such an externality cannot be inferred solely from the observation that behavior has diffused within a household.

### 4.9. Integrated Conceptual Framework

Taken together, the proposed framework distinguishes the mechanisms governing individual adaptation, household diffusion, and downstream consequences.

The first stage proposes that individual behavioral adaptation is influenced by trigger clarity, behavioral controllability, and feedback immediacy:

(TC,BC,FI)→BA.

The second stage proposes that the translation of individual adaptation into household behavioral spillover depends additionally on behavioral visibility, environmental sharedness, and household relevance:

$$BA\underset{BV,\;ES,\;HR}{\to }BS.$$

However, because household members may initiate, facilitate, reinforce, or resist disease management behaviors, patient and household responses are conceptualized as reciprocal:

BA↔BS.

Finally, the consequences of household behavioral spillover are left empirically open rather than assumed to be beneficial:

$$BS\to \left\{\mathrm{positive},\;\mathrm{negative},\;\mathrm{mixed},\;\mathrm{or}\;\mathrm{null}\;\mathrm{consequences}\right\}.$$

Figure 1 summarizes these relationships. The framework distinguishes clinical disease characteristics from their behavioral appraisal, individual adaptation from interpersonal diffusion, and behavioral diffusion from its eventual health and welfare consequences. It thereby provides a structure within which the illustrative dermatological conditions considered in this article can generate testable hypotheses without treating the proposed disease classifications as empirically established facts.

### 4.10. Empirical Implications and Testable Propositions

The revised framework generates several propositions that can guide future empirical investigation.

**Proposition 1.** 
*Greater trigger clarity, behavioral controllability, and feedback immediacy are expected, individually or interactively, to be associated with stronger individual behavioral adaptation.*

**Proposition 2.** 
*Conditional on individual behavioral adaptation, household behavioral spillovers are expected to be greater when disease management behaviors are more visible, occur within more strongly shared environments, and are more relevant to other household members.*

**Proposition 3.** 
*Individual behavioral adaptation is neither a necessary nor a sufficient condition for household behavioral adaptation. Household behavior may precede patient adaptation, particularly in caregiver-mediated disease management, while strong patient adaptation may remain individual when opportunities for interpersonal diffusion are limited.*

**Proposition 4.** 
*Household responses may reinforce or inhibit subsequent patient behavioral adaptation, generating reciprocal rather than strictly unidirectional relationships between patient and household behavior.*

**Proposition 5.** 
*The occurrence and magnitude of behavioral spillover do not determine the direction of its consequences. Similar degrees of behavioral diffusion may generate positive, negative, mixed, or null health and welfare effects across different patients and households.*

These propositions are intended to make the framework empirically falsifiable rather than to describe relationships already established in the dermatology literature. Future studies could operationalize TC, BC, and FI using clinical assessments, patient perceptions, or both, while BV, ES, and HR could be measured using patient-caregiver or household-level data. Longitudinal designs following patients and household members from diagnosis or treatment initiation would be particularly useful for distinguishing behavioral changes associated with disease management from pre-existing shared household practices.

Because patient and household behaviors are unlikely to be statistically independent, dyadic or household-level designs may be preferable to analyses based exclusively on individual patients. Such studies could also examine whether the proposed relationships vary according to age, disease severity, socioeconomic resources, caregiver involvement, household composition, and cultural setting.

## 5. Discussion

The present article develops a conceptual framework for understanding how disease management behaviors may extend beyond individual patients and become embedded in household behavior. Its central contribution is to distinguish two analytically related but separate processes: individual behavioral adaptation and household behavioral diffusion. At the individual level, trigger clarity, behavioral controllability, and feedback immediacy are proposed as determinants of the likelihood and intensity of behavioral adaptation. At the household level, behavioral visibility, environmental sharedness, and household relevance are proposed as conditions that influence whether these adaptations extend beyond the patient. This distinction addresses an important limitation of approaches that treat behavioral adaptation and interpersonal diffusion as a single process.

The framework is consistent with, but not reducible to, established theories of health behavior. The Health Belief Model, the Theory of Planned Behavior, and Social Cognitive Theory emphasize constructs such as perceived susceptibility, expected benefits and barriers, self-efficacy, perceived behavioral control, environmental influences, and observational learning. The present framework draws on these traditions but reorganizes relevant mechanisms around the specific question of how disease management behavior may develop at the patient level and subsequently become relevant to other household members. In this sense, trigger clarity, behavioral controllability, and feedback immediacy should be interpreted as a conceptual synthesis rather than as substitutes for existing behavioral theories.

A second contribution concerns the distinction between behavioral spillovers and economic externalities. Changes in the behavior of family members following another household member’s diagnosis or disease management experience represent behavioral diffusion, but they do not necessarily constitute economic externalities. Household decisions may reflect altruism, caregiving, shared preferences, collective purchasing, common resource constraints, or joint health production. Whether a behavioral spillover creates an externality therefore depends on whether the resulting benefits or costs experienced by others are internalized within the relevant decision-making process. Maintaining this distinction is particularly important when applying health–economic concepts within families.

The framework also emphasizes that household spillovers should be treated as directionally neutral. Behavioral diffusion may generate beneficial outcomes when it promotes appropriate preventive practices, facilitates treatment adherence, reduces relevant exposures, or improves the shared environment. However, household responses may also be costly, burdensome, unnecessarily restrictive, or anxiety-driven. Mixed outcomes are possible when the same behavioral change benefits the patient while imposing financial, psychological, or lifestyle costs on other household members, and behavioral diffusion may sometimes occur without measurable health consequences. Consequently, the occurrence of behavioral spillover and the welfare consequences of that spillover should be investigated as separate empirical questions.

An additional implication of the framework is that patient and household behavior should not necessarily be understood as a unidirectional sequence. In caregiver-dependent populations, particularly children, older adults, and other individuals requiring assistance with disease management, household adaptation may precede meaningful patient-level behavioral adaptation. Parents or caregivers may select products, modify environmental conditions, organize treatment routines, or alter shared activities independently of the patient’s own behavioral response. More generally, household behavior may reinforce, facilitate, resist, or weaken subsequent patient adaptation. The relationship is therefore better represented as a process of patient–household co-adaptation than as a simple progression from disease characteristics to patient behavior and then to household behavior.

The coordination game developed in this article formalizes one specific mechanism through which such interdependence may arise. Where the effectiveness or net benefit of one party’s behavioral adaptation depends partly on compatible behavior by another household member, strategic complementarity may generate multiple equilibria. Coordinated adoption may be mutually beneficial while nevertheless failing to occur if each party expects the other not to adopt. At the same time, the coordination model should not be interpreted as a complete representation of household behavioral spillovers. Observational learning, caregiver initiative, social influence, shared environments, and common routines may generate behavioral diffusion even in the absence of strategic interaction.

Several boundary conditions should therefore be recognized. First, the proposed dimensions are not fixed characteristics of particular diagnoses. The same disease may be perceived differently across patients depending on age, disease severity, previous experience, health literacy, clinician communication, socioeconomic resources, treatment access, household composition, and cultural context. Trigger clarity, behavioral controllability, and feedback immediacy may therefore reflect both objective clinical characteristics and patients’ perceptions of those characteristics. Likewise, behavioral visibility, environmental sharedness, and household relevance may vary substantially across households even when the underlying diagnosis is identical.

Second, the four dermatological conditions considered in this article are illustrative rather than exhaustive or representative. Their purpose is to demonstrate how different combinations of clinical and behavioral characteristics may generate contrasting expectations within the framework. They should not be interpreted as occupying fixed positions on a universal behavioral-spillover scale. Empirical research is required to determine whether the proposed tendencies are observed in practice and how strongly they vary across patient populations, disease subtypes, and household contexts.

Third, the framework is intentionally conceptual. It does not estimate the magnitude or causal effects of behavioral spillovers. Future studies could operationalize trigger clarity, behavioral controllability, and feedback immediacy using clinical assessments, patient-reported perceptions, or combinations of both. Household-level constructs could be measured through patient–caregiver dyads, family surveys, household behavioral observations, or repeated measures of shared routines and environmental practices. Longitudinal studies beginning at diagnosis or treatment initiation may be particularly useful for distinguishing disease-related behavioral change from pre-existing household behavior. Dyadic and household-level analytical approaches may also be preferable to individual-level models because patient and household responses are unlikely to be statistically independent.

The conceptual implications may also extend beyond dermatology. The four dermatological conditions used here should be viewed as illustrations of mechanisms that may operate in other diseases when disease management affects caregivers, family members, shared environments, or household routines. The importance of lifestyle modification in cardiovascular disease [21], for example, illustrates that disease-related behavioral adaptation is not unique to dermatology. At a broader health–economic level, Claypool et al. [2] demonstrate, in the context of chikungunya and dengue control, that health interventions may generate positive health externalities extending beyond directly targeted individuals. Although infectious-disease externalities operate through mechanisms different from the household behavioral spillovers considered here, both perspectives emphasize that the consequences of disease management need not terminate with the focal patient. Whether the specific behavioral mechanisms proposed in the present framework generalize across disease categories remains an empirical question.

From a health–economic perspective, this broader application suggests that the relevant consequences of disease management may not be captured fully by focusing only on the treated individual or on total costs to society.

## 6. Conclusions

This article proposes a conceptual framework for distinguishing individual behavioral adaptation from household behavioral spillovers in chronic disease management. At the patient level, trigger clarity, behavioral controllability, and feedback immediacy are proposed as determinants of behavioral adaptation. At the household level, behavioral visibility, environmental sharedness, and household relevance are proposed as conditions influencing whether disease management behaviors extend beyond the patient. The framework additionally allows reciprocal patient–household adaptation and treats the consequences of behavioral spillovers as potentially positive, negative, mixed, or null.

The dermatological conditions considered in this article serve as illustrative settings rather than as empirically established rankings of behavioral-spillover potential. Their purpose is to demonstrate how variation in disease characteristics, patient perceptions, caregiving requirements, and household contexts may generate different behavioral pathways. The proposed relationships therefore represent testable hypotheses rather than established causal effects.

Future research should evaluate these propositions using longitudinal, dyadic, and household-level data and determine whether accounting for behavioral effects on close household members improves the understanding of disease management, resource allocation, and aggregate welfare. More broadly, the framework suggests that the consequences of chronic disease management may extend beyond the treated individual and that these wider behavioral effects warrant explicit empirical investigation.

## Figures and Tables

**Figure 1:**
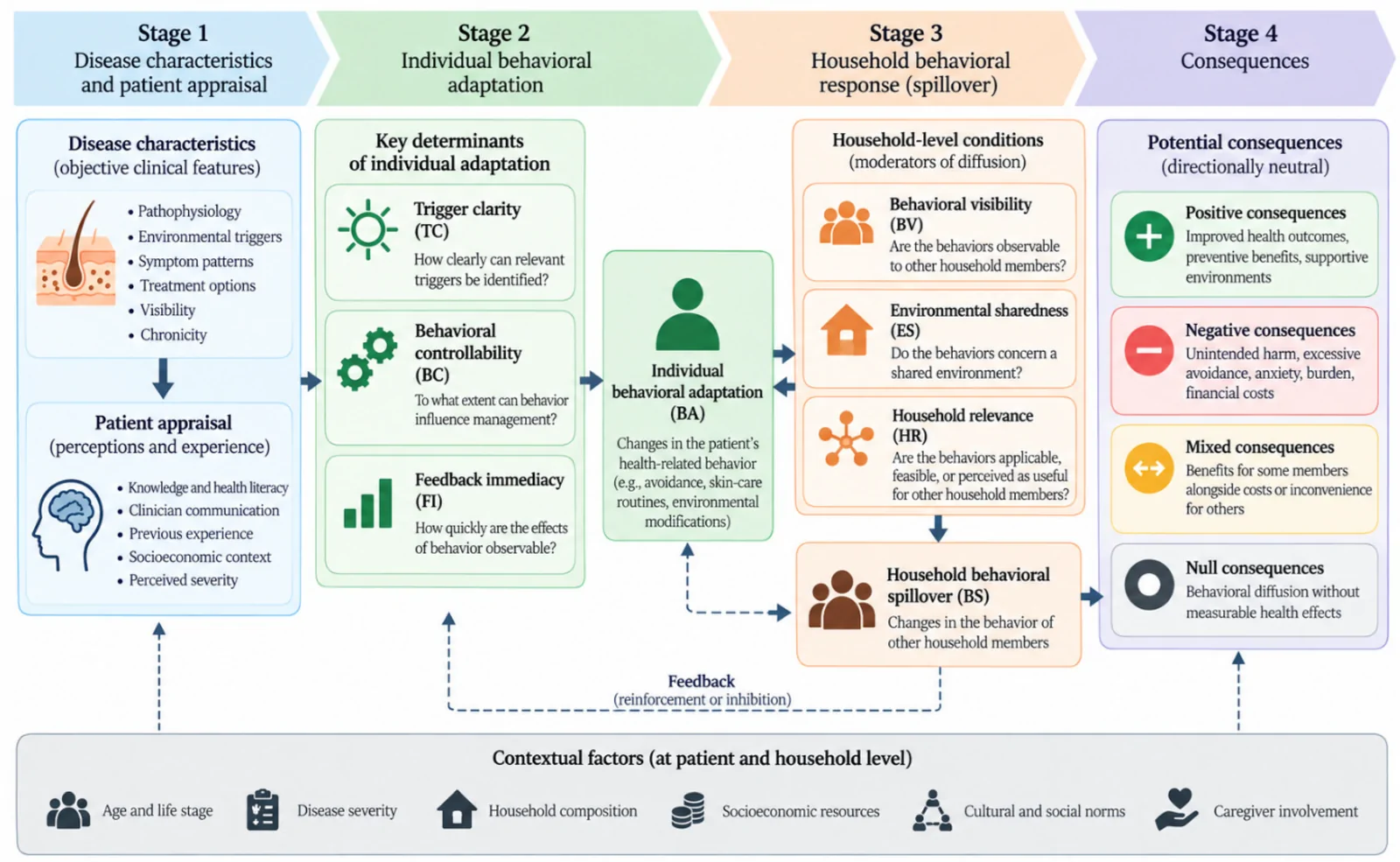
Integrated conceptual framework for individual behavioral adaptation and household behavioral spillovers in dermatological diseases. Note: This figure illustrates the proposed conceptual framework. Disease characteristics influence patient appraisal, which in turn shapes individual behavioral adaptation (BA) through trigger clarity (TC), behavioral controllability (BC), and feedback immediacy (FI). Household behavioral spillovers (BS) are influenced by individual behavioral adaptation and moderated by behavioral visibility (BV), environmental sharedness (ES), and household relevance (HR). The relationship between individual and household behavioral adaptation is bidirectional, allowing household responses to reinforce or inhibit patient behavior and, in some circumstances, to precede patient-level adaptation. Behavioral spillovers are treated as directionally neutral and may generate positive, negative, mixed, or null consequences. Contextual factors—including age and life stage, disease severity, household composition, socioeconomic resources, cultural and social norms, and caregiver involvement—may influence the relationships throughout the framework.

**Table 1:** Illustrative application of the conceptual dimensions to selected dermatological conditions.

Condition	Trigger Clarity	Behavioral Controllability	Feedback Immediacy	Household Diffusion Pathway
Photosensitivity disorders	Relatively high for identifiable UV exposure	Relatively high for exposure-reduction behaviors	Variable	Visible/shared photoprotection
Atopic dermatitis	Variable	Variable/moderate	Variable	Strong caregiver and household-environment component
Psoriasis	Often lower/less direct	Limited/variable	Often less immediate	Lifestyle/shared routines possible but not inherent
Acne	Variable	Moderate for treatment adherence	Variable	Often predominantly individual routines

Note: These classifications represent illustrative theoretical tendencies rather than empirical rankings or fixed properties of the diagnoses. Considerable heterogeneity is expected across patients, disease subtypes, severity levels, and social contexts.

**Table 2:** Patient–Household Coordination Game: General Payoff Structure.

	Household: Adopt (A)	Household: Not Adopt (N)
**Patient: Adopt (A)**	(B−C, B−C)	$\left(\alpha ,B-C\;0\right)$
**Patient: Not Adopt (N)**	$\left(0,\;\alpha B-C\right)$	$\left(0,\;0\right)$

Note: Payoffs are reported as (Patient, Household). B denotes the benefit to each player under coordinated adoption, C denotes the individual cost of adoption, and 0≤α<1 denotes the fraction of the coordinated benefit obtainable through unilateral adoption. The payoffs are theoretical utilities rather than empirically estimated monetary or health values.

**Table 3:** Patient–household coordination game: numerical illustration.

	Household: Adopt (A)	Household: Not Adopt (N)
**Patient: Adopt (A)**	**(3, 3)**	**(−1, 0)**
**Patient: Not Adopt (N)**	**(0, −1)**	**(0, 0)**

Note: Payoffs are reported as (Patient, Household). The numerical values are illustrative and are not empirically estimated. The example assumes B=5, C=2, and α=0.20, satisfying the coordination-game condition αB<C<B. Thus, coordinated adoption yields a payoff of B−C=3 to each player, whereas unilateral adoption yields αB−C=−1 to the adopting player.

## Data Availability

No new data were created or analyzed in this study. Data sharing is not applicable to this article. This article is a conceptual/theoretical paper and does not involve the generation, collection, or analysis of original research data. The manuscript develops a conceptual framework based exclusively on previously published literature and therefore does not produce a new dataset that could be deposited in a public repository.
